# Retraction: Zinc biofortification potential of diverse mungbean [*Vigna radiata* (L.) Wilczek] genotypes under field conditions

**DOI:** 10.1371/journal.pone.0277596

**Published:** 2022-11-16

**Authors:** The PLOS ONE Editors

The *PLOS ONE* Editors retract this article [1] because it was identified as one of a series of submissions for which we have concerns about authorship, competing interests, and peer review. We regret that the issues were not addressed prior to the article’s publication.

MF, SUA, and SF did not agree with the retraction. MUH, MH, MJA, and MSA either did not respond directly or could not be reached.

## References

[1] Haider MU, Hussain M, Farooq M, Ul-Allah S, Ansari MJ, Alwahibi MS, et al. (2021) Zinc biofortification potential of diverse mungbean [*Vigna radiata* (L.) Wilczek] genotypes under field conditions. PLoS ONE 16(6): e0253085. 10.1371/journal.pone.0253085 34161364 PMC8221514

